# Clinical use of whole genome sequencing for *Mycobacterium tuberculosis*

**DOI:** 10.1186/s12916-016-0598-2

**Published:** 2016-03-23

**Authors:** Adam A. Witney, Catherine A. Cosgrove, Amber Arnold, Jason Hinds, Neil G. Stoker, Philip D. Butcher

**Affiliations:** Institute of Infection and Immunity, St George’s University of London, Cranmer Terrace, London, SW17 0RE UK; Clinical Infection Unit, St George’s University Hospitals NHS Foundation Trust, Blackshaw Road, London, SW17 0QT UK

**Keywords:** Whole genome sequencing, Multidrug resistance, Tuberculosis

## Abstract

Drug-resistant tuberculosis (TB) remains a major challenge to global health and to healthcare in the UK. In 2014, a total of 6,520 cases of TB were recorded in England, of which 1.4 % were multidrug-resistant TB (MDR-TB). Extensively drug-resistant TB (XDR-TB) occurs at a much lower rate, but the impact on the patient and hospital is severe. Current diagnostic methods such as drug susceptibility testing and targeted molecular tests are slow to return or examine only a limited number of target regions, respectively. Faster, more comprehensive diagnostics will enable earlier use of the most appropriate drug regimen, thus improving patient outcomes and reducing overall healthcare costs. Whole genome sequencing (WGS) has been shown to provide a rapid and comprehensive view of the genotype of the organism, and thus enable reliable prediction of the drug susceptibility phenotype within a clinically relevant timeframe. In addition, it provides the highest resolution when investigating transmission events in possible outbreak scenarios. However, robust software and database tools need to be developed for the full potential to be realized in this specialized area of medicine.

## Background

Early observations made in 2008 [1] predicted the potential for whole genome sequencing (WGS) to transform tuberculosis (TB) diagnostics, when the then recent use of “next generation” sequencing technology to analyse extensively drug-resistant (XDR) isolates of *Mycobacterium tuberculosis* was summarized. Such has been the speed of technological advances that the description of 1 gigabase (Gb) of sequence as “staggering” in 2008 is now dwarfed by the current state-of-the-art machines, producing up to 15 Gb on the benchtop MiSeq and 1,500 Gb on HiSeq 4000. The inevitable economies of scale have therefore led to a rapid drop in costs, thus opening up microbiology diagnostics to direct sequencing applications.

In order to consider the potential for WGS to improve diagnosis, treatment and management of TB in a clinical setting, it is useful to understand the current diagnostic workflow. The exact details of this process (and thus the potential impact of WGS) vary between different settings and countries, so we discuss this in the context of St George’s Hospital, London, UK. In 2014, a total of 6,520 cases of TB (incidence of approximately 12 per 100,000) were recorded in England, of which 1.4 % were multidrug-resistant TB (MDR-TB); defined as resistant to isoniazid (INH) and rifampicin (RIF) [2]. However, both TB (30 per 100,000) and MDR-TB levels are higher in London. Extensively drug-resistant TB (XDR-TB) occurs at a much lower rate, but the requirements for effective clinical management and treatment of the MDR-TB and XDR-TB and the public health management of such cases are even more onerous.

### Current practice

The standard procedure for a case of TB at St George’s Hospital would be as follows. A patient is referred from a primary care setting, a chest clinic or a local hospital as having suspected pulmonary TB, or attends of their own volition. A sputum sample is taken, examined by microscopy for acid-fast bacilli (AFB) and a BacT/Alert (Organon Teknika Corporation, Durham, NC, USA) [3] liquid culture inoculated. If TB is clinically suspected then treatment will be started immediately; otherwise, the smear results will be returned within 1–2 days, and if positive, the patient is started on standard TB treatment according to WHO guidelines [4, 5].

If the *M. tuberculosis* is fully sensitive, this is the correct treatment regimen. However, there is a significant chance that antibiotic resistance may be present, and if true, the standard treatment may be completely ineffective, or if only partially so, could make the development of resistance more likely. Therefore, when BacT/Alert cultures become positive, usually within 1–2 weeks, they are sent to the UK’s national reference laboratory (NRL) for first-line drug phenotypic susceptibility testing (DST). Results are returned within 2–3 weeks, but if evidence of resistance is found then second-line DST is begun, returning after a further 2–3 weeks. It can therefore take up to 8 weeks after presentation before a full DST profile is available for resistant cases.

In order to speed up this process, St George’s Hospital carries out molecular tests to identify likely MDR-TB cases. Following microscopy, an AFB smear-positive sputum is also tested for genetic markers of RIF resistance using the Xpert® MTB/RIF assay (Cepheid, Sunnyvale, CA, USA) [6]; results are available within two days. Other hospitals may wait for a positive culture before performing an Xpert® MTB/RIF assay, with the subsequent delays that may then occur. A negative Xpert® MTB/RIF assay result for RIF resistance leads to continuation of standard drug-sensitive treatment, with subsequent public health action to trace contacts, assess exposure with Mantoux skin tests and offer INH prophylaxis, where appropriate. A positive Xpert® MTB/RIF assay result indicating RIF resistance suggests that this is now a likely case of MDR-TB, an assumption that has considerable clinical and public health consequences. It leads to an assessment of the risk factors for resistance and the clinical condition of the patient, in order to decide whether to cease first-line drug treatment and switch to a second-line drug regimen [4, 5] at this stage or wait for further molecular confirmation of resistance. Furthermore, heightened infection control measures are implemented and extended public health questionnaires initiated.

Other molecular tests are available, notably the GenoType MTBDR*plus* (Hain Lifescience, GmbH, Nehren, Germany) [7] which identifies selected markers for INH resistance in addition to RIF, further confirming MDR-TB, and the GenoTypeMTBDR*sl* (Hain Lifescience) [8] which also identifies selected resistance markers for fluoroquinolones, aminoglycosides and ethambutol, indicative of XDR-TB. In our context, these tests are available from the NRL but have to be specifically requested.

WGS has two main overlapping uses in clinical microbiology and public health: i) identification of genotypes which can be used to predict drug-resistant phenotype; and ii) the determination of genetic relatedness which can identify transmission chains in potential outbreak scenarios. Both of these can have direct patient benefits.

### Genotype–phenotype prediction

The prediction of antibiotic resistance phenotype from genotype is not yet a solved problem; however, it is a more straightforward task for *M. tuberculosis* than for other species due to a lack of horizontal gene transfer. Indeed, drug resistance is generally thought to be mediated only through mutations in specific gene targets. The key, therefore, is to identify those single nucleotide polymorphisms (SNPs) which are responsible for, or strongly associated with, resistance.

Many studies have been performed identifying individual markers of resistance; however, more recently, several large sequencing studies have analysed clinical isolates and correlated SNPs to phenotypic drug sensitivity results, calculating predictive sensitivities and specificities of sets of SNPs [9–11]. In this context, specificity describes the likelihood that SNPs identified lead to resistance, while sensitivity describes the proportion of resistant cases that are identified through that set of SNPs. But the confidence for which a prediction can be made has been shown to vary according to not only drug class but also different members of these classes. This is slightly complicated by the fact that the gold standard (culture) has its own error rate [12].

The aforementioned molecular diagnostic tests are targeted methods for predicting the phenotype of the organism from the detected genotype. These examine a limited number of sites known to be associated with varying levels of resistance to the specific drugs mentioned. False negatives can arise, for example, when any rifampicin-resistant (RIF^R^) mutations that lie outside the *rpoB* RIF resistance-determining region (RRDR), such as the I491F mutation in RpoB [13, 14], are not detected by the Xpert® MTB/RIF assay leading to the isolate being incorrectly called susceptible. Similarly, false positives can arise when a polymorphism in the RRDR region of *rpoB* is detected and RIF^R^ predicted when they are either completely rifampicin-sensitive (RIF^S^) or have a low level of resistance that could be overcome by an appropriately increased dosage (see below). Furthermore, it is important to understand that resistance is not a binary phenomenon, and the variable impact of individual mutations on the minimum inhibitory concentration (MIC) raises the possibility of designing personalized treatment regimes, for example:

i) It has been established that INH resistance, predominantly mediated through loss of catalase-peroxidase activity via mutations in *katG*, produce high-level resistant strains (MIC: 2–8 μg/ml INH) and that the KatG S315T substitution occurs in 50–95 % of INH-resistant isolates [15]. However, mutations in the *inhA* gene or promotor region produce low-level resistant strains (MIC: 0.2–0.5 μg/ml) occurring in 8–43 % of INH-resistant isolates. Therefore, if a *katG* high-level resistance mutation is detected, isoniazid therapy should not be included and could be substituted with prothionamide. Prothionamide has the same drug target as INH, namely InhA, but it is activated from the pro-drug form to the active drug by a different enzyme, EthA, and mutations in the *ethA* gene might contraindicate the use of prothionamide. If, however, an *inhA* promotor mutation is present suggesting low-level resistance, then INH could remain in the regimen but at a higher dosage as INH has a high therapeutic margin.
ii) Moxifloxacin is a second-line drug included in standard MDR-TB regimens, and specific mutations in the *gyrA* gene confer resistance often suggesting the presence of an XDR-TB isolate. However, different mutations in *gyrA* impact MIC values differently according to the quinolone type [16]. For example, an MIC value for moxifloxacin of 1 μg/mL has been shown in isolates with the A90V substitution in GyrA. This would be called resistant by conventional phenotyping using the standard breakpoint of >0.5 μg/ml, but Feasey et al. [17] showed successful treatment by increasing the dose of moxifloxacin from 400 to 600 mg in order to achieve in vivo drug levels above the MIC. Subsequent identification of this A90V mutation in other clinical isolates could be used as evidence to indicate similar dosage alterations for effective treatment in the face of a resistant DST result.
iii) Mutations in the 89 base pair RRDR of *rpoB* confer a range of resistance phenotypes with large variations in MICs [18, 19]. Knowing the exact mutation and subsequent amino acid change is therefore crucial. A case of possible MDR-TB could be indicated by a positive Xpert® MTB/RIF assay and called RIF^R^ because of a SNP in the RRDR that is different from wild-type, identified by one of the five probes used in that test. This SNP could be a simple phylogenetic polymorphism independent of resistance, or a SNP associated with low-level RIF^R^. Such a false positive result would wrongly indicate MDR-TB and exclusion of RIF in the initial regimen prior to phenotypic susceptibility testing. For example, MIC data for a range of non-synonymous SNPs at codon 526 has been reported ranging from 1–256 μg/ml [18, 19]. Thus, RpoB H526N, an allele we recently found in a *M. tuberculosis* patient isolate, has an MIC of 0.25 μg/ml, so rifampicin can still be included in the regimen despite the Xpert® MTB/RIF assay returning a resistant result at initial diagnosis. Furthermore, different alleles have different levels of cross-resistance to rifabutin (RFB), a semi-synthetic derivative of RIF [19, 20]. For example, mutation H526L is associated with a RIF^R^/RFB^S^ pattern and might inform the substitution of RFB into a therapy regimen. Sequence data might thus direct the use of either RIF or RFB depending on the exact allele identified.

A recent and comprehensive report from the TBNET and RESIST-TB networks collated genotype–phenotype data from literature and electronic databases and reached a consensus about reporting standards in the clinical use of molecular DST results [21]. The report summarised the clinical implications of mutations detected by molecular methods and provided much needed clarity on the issue of how genotypic data can be interpreted in terms of TB drug regimes.

### Determination of genetic relatedness

In the UK, outbreak identification is currently determined using the mycobacterial interspersed repetitive unit-variable number tandem repeat (MIRU-VNTR) method [22], which can suggest clusters of isolates but lacks the resolution to be certain of possible transmission events. The identification of such events clearly has a role in public health intervention, but can also directly benefit the patient if clinical outcome data is available for the strain in question, ensuring that patients and infected contacts can be isolated and initiated on the most effective treatment as quickly as possible. We have experienced this scenario at St George’s Hospital; WGS identified an XDR-TB strain from a hospital in-patient with smear-positive pulmonary TB and no risk factors of MDR/XDR-TB, as identical to a known XDR-TB strain that we had previously sequenced. The patient was switched to an XDR-TB regimen and subsequent sputum samples showed culture conversion.

Several studies have attempted to validate WGS for public health interventions. Gardy et al. [23] demonstrated the limited resolution of MIRU-VNTR for outbreak investigation by sequencing 32 *M. tuberculosis* isolates from a threeyear outbreak of TB in British Columbia, Canada. WGS data clearly showed the presence of two distinct lineages of *M. tuberculosis* with identical MIRU-VNTR types, although they had both likely descended from a historical common ancestor. Furthermore, the integration of WGS with social network information identified several transmission events and the presence of super-spreaders, leading to the conclusion that the outbreak coincided with a recorded increase in crack cocaine usage.

Török et al. [24] investigated two cases of MDR-TB associated with a language school in Cambridge, UK. The cases had an epidemiological link through the school, but there was a discrepancy in phenotypic results; one was determined streptomycin-resistant and the other sensitive. The UK’s National Mycobacterial Reference Laboratory showed that both isolates were of the Beijing lineage and of the same MIRU-VNTR type; however, the phenotypic difference suggested that they were different strains. Subsequent WGS showed that the isolates did not display any differences in SNPs, suggesting a missed direct or indirect transmission event. This case highlights a particular problem with the reliability of phenotypic testing, and the authors suggest that MICs close to the breakpoint for streptomycin could have affected the reproducibility and thus the discrepancy between the two isolates.

Walker et al. [25] undertook a larger, retrospective observational study using WGS to delineate TB clusters within 390 isolates from 254 patients archived over a 17-year period from the central UK region. By analysing the patterns of mutations the study enabled an inference about direction of transmission within outbreaks and also identified the existence of potential super-spreaders in several of the clusters investigated.

Pankhurst et al. [26] identified several outbreak scenarios which had been previously missed by standard routine methods, and demonstrated that WGS in routine use can identify transmission networks and direct better public health interventions.

### Clinical consequences

Clinical management of antibiotic-resistant TB is complex. The drugs are often poorly tolerated, and side-effects include ototoxicity, nephrotoxicity, hepatitis, diarrhoea, vomiting and psychological disturbances [27]. Additionally, the requirement for an injectable drug in the regime requires the insertion of a central long line, which exposes the patient to an increased risk of infection and venous thrombosis. According to UK national guidelines [28], patients should be isolated in negative pressure, HEPA-filtered single occupancy rooms until 3 weekly negative smears have been obtained that are ideally also culture negative. At St George’s Hospital we wait for the repeated respiratory samples to be culture-negative after a 6 week incubation. These measures are unpleasant for the patient, expensive for the hospital and compliance can be difficult. Where drug resistance is suggested, second-line MDR treatment is started in a complete absence of detailed resistance information, and DST results only appear up to 5–8 weeks after presentation. In our experience, the initial regimen is often not optimal, and when XDR-TB cases are encountered may be completely ineffective. Even when optimal, it may be desirable to change because of drug side-effects, and to do this in the absence of resistance information is not ideal.

The routine use of a genotypic RIF testing gives a faster indication of the suitability of the drug regimen. In settings where this is not done, DST for MDR-TB cases will be carried out in two phases, leading to further delays. In these cases, patients are likely to have spent the first 3–5 weeks on ineffective first-line drugs, and will only have generic MDR treatment for the subsequent 2–3 weeks.

False negative and false positive tests for resistance have significant implications for the patient. Incorrectly assigning a patient to drug-sensitive TB means many weeks of infective treatment and subsequent further resistance developing, whilst incorrectly assigning to a regime for drug-resistant TB has implications due to the drug toxicities and stringent public health measures implemented. The consequences of such errors are severe, especially when it is considered that these will only be corrected once the full DST profile is received, up to 8 weeks later. WGS avoids these specific problems by presenting an unbiased view of the whole genome; all sites are examined simultaneously, in a timeframe considerably shorter than current methods. WGS interpretation does, however, require expertise in bioinformatics and data analysis tools that may not be available in all settings.

Several studies have investigated the potential impact of WGS on patient benefit by attempting to predict drug susceptibilities from the isolate genotypes [26, 29–31]. Witney et al. [29] demonstrated that WGS can be used to provide treating physicians with valuable information in a clinically relevant timeframe, several weeks before DST results can be returned. More recently, Pankhurst et al. [26] demonstrated routine use of WGS and calculated that the system cost £481 (WGS alone), £518 (DST alone) or £540 (DST and WGS), demonstrating that WGS can be economically viable. Furthermore, these differences in cost are tiny when compared to the costs of in-patient treatment for drug-resistant TB; at St George’s Hospital, we estimate that treating a TB patient in the airflow-controlled ward costs £900 per day, not including the cost of the often expensive drugs required for MDR and XDR cases. The case for any intervention that has the potential to save weeks of patient care is therefore clear.

All the studies described so far have applied WGS to cultured isolates; however, a further 1–2 weeks could be saved if the culture step is eliminated. WGS applied direct to sputum is a difficult problem due to the low number of organisms and the presence of contaminating human and non-mycobacterial DNA. To address this problem, Brown et al. [32] used DNA sequence capture with biotinylated RNA bait oligonucleotides, spanning the entire *M. tuberculosis* genome, followed by amplification and sequencing of the captured DNA. This method was applied to 24 TB sputum specimens and showed good on-target reads and depth of coverage for 23 samples with associated accurate prediction of drug resistance mutations. Evidence for a mixed infection with two strains in one sample was also predicted from the genome sequence data and this indicates another potential read-out of WGS that is unlikely to be obtained through other methods. Such direct use of WGS has been proposed as the basis for personalizing therapy for drug-resistant TB [33].

WGS is not a stationary technology [34]; development of miniaturized sequencing platforms such as the MinION from Oxford Nanopore Technologies (Oxford, UK) will eventually democratize the implementation of WGS in the clinical management of TB by substantially reducing the cost, making the economic, technical and clinical arguments for WGS implementation incontrovertible [35].

### Bioinformatics

Bioinformatic analysis is a key part of the WGS process, and for optimal clinical use, issues within the analysis pipelines and database resources need to be addressed.

The analysis process can be broken down into three steps: i) alignment of the DNA sequence data to a reference genome, normally the H37Rv strain of *M. tuberculosis*; ii) variant site calling which determines the probability that a SNP is correctly identified in the sequence and is not due to a random error introduced during the sequencing reaction; and iii) resistance prediction or phylogenetic reconstruction. Numerous alignment tools are available and all have strengths and weaknesses [36], such that the choice is invariably made by personal preference. Variant site calling commonly involves either SAMtools [37], FreeBayes [38] or GATK [39], and resistance prediction is often implemented using custom software, comparing identified mutations with databases of known resistance-conferring SNPs. Phylogenetic reconstruction from sequence data is not a new problem, but when applied to clinical WGS data some work still needs to be done with regards to estimating the error when determining SNP number differences between isolates in potential transmission chains; the identification of a transmission event can have significant downstream clinical and public health impact.

Many software pipelines have been developed and are in use in individual laboratories or available via web interfaces [9, 39]. However, the future implementation of WGS in clinical practice will depend on the development of robust and validated software tools that are easy to use and interpret in the diagnostic context. Promising examples are under development that can predict a phenotype within minutes of uploading sequence data, for example Mykrobe [40]. Efforts are also under way to assess and standardize methods, for example the Global Microbial Identifier group [41] initiated an open proficiency test in 2015, providing standard DNA material or sequence data for processing by participants. Although this does not currently include *M. tuberculosis*, the alignment and site calling steps described above are generally organism-independent. However, such standard reference data are essential to develop consensus, thus encouraging better inter- and intra-laboratory reproducibility.

The reliability of the genotype–phenotype prediction is dependent not only on good correlation data but also on good database resources to allow easy access to such data [42]. TBDReaMDB [43] was the first TB-specific database of drug resistance-conferring mutations, created in 2009, and more recently updated in 2014. It was collated through literature searches; however, it now accepts submissions from external users. TBDReaMDB does not attempt to validate target mutations, instead only cataloguing studies examining mutations and genes of interest. For genotyping by WGS to become effective, validated and continuously updated databases need to be developed and made available; successful examples include the public HIV Drug Resistance Database [44]. The recently funded ReSeqTB project [45], which aims to implement a single repository for validated TB drug-resistant correlations, may fulfil this role; however, it is still in the early stages of development. Furthermore, it is interesting to note that the aforementioned HIV database also includes data on genotype-clinical outcome correlations. Maybe this would be a useful addition to any TB mutation repository, since sharing successful treatment options for already sequenced strains could be as effective as predicting phenotype solely from genotype. The mechanics of the interaction between the software tools and global databases of resistance mutations and phylogenetic information is yet to be determined. However, the true power of WGS will not be realised unless open and integrated systems are put in place.

Arguably WGS could be used to expand the current rapid, targeted molecular methods by improving the predictive power of any identified mutation, but whereas the targeted molecular approach requires constant technical development to maintain a comprehensive mutation detection list, WGS requires only updates to its predictive algorithm, thus improving the diagnostic power more rapidly.

## Conclusions

The advantages of WGS in the TB diagnostic workflow are clear when compared to standard DST; results can be returned several weeks earlier and, in the future, this will only become faster and cheaper. Compared to current molecular tests, until WGS can be routinely performed on sputum, results will lag by 1–2 weeks. Nevertheless, the vastly increased resolution of WGS results means a more accurate and comprehensive picture of resistance prediction than is currently possible.

In addition, the ability to inform transmission detection, without the need for extra tests, and the comparability of cost ensure that the use of WGS in clinical microbiology is just a matter of how and when, rather than if. Current indications are that dual testing (WGS and DST) is only marginally more expensive than WGS alone, and while certainty in phenotypic resistance is not yet (if at all) possible, it would be prudent to maintain DST in the standard workflow. More research focusing on the realities of clinical management and economic benefits and constraints in different environments will be immensely valuable.

## Abbreviations

AFB: acid-fast bacilli
DST: drug susceptibility testing
INH: isoniazid
MDR-TB: multidrug-resistant tuberculosis
MIC: minimum inhibitory concentration
MIRU-VNTR: mycobacterial interspersed repetitive unit-variable number tandem repeat
NRL: national reference laboratory
RFB: rifabutin
RIF: rifampicin
RRDR: rifampicin resistance-determining region
SNP: single nucleotide polymorphism
TB: tuberculosis
WGS: whole genome sequencing
WHO: World Health Organization
XDR-TB: extensively drug-resistant tuberculosis
